# Frataxin Deficiency Promotes Excess Microglial DNA Damage and Inflammation that Is Rescued by PJ34

**DOI:** 10.1371/journal.pone.0151026

**Published:** 2016-03-08

**Authors:** Yan Shen, Marissa Z. McMackin, Yuxi Shan, Alan Raetz, Sheila David, Gino Cortopassi

**Affiliations:** 1 Department of Molecular Biosciences, University of California Davis, Davis, California, 95616, United States of America; 2 Department of Chemistry, University of California Davis, Davis, California, 95616, United States of America; Massachusetts General Hospital and Harvard Medical School, UNITED STATES

## Abstract

An inherited deficiency in the frataxin protein causes neurodegeneration of the dorsal root ganglia and Friedreich's ataxia (FA). Frataxin deficiency leads to oxidative stress and inflammatory changes in cell and animal models; however, the cause of the inflammatory changes, and especially what causes brain microglial activation is unclear. Here we investigated: 1) the mechanism by which frataxin deficiency activates microglia, 2) whether a brain-localized inflammatory stimulus provokes a greater microglial response in FA animal models, and 3) whether an anti-inflammatory treatment improves their condition. Intracerebroventricular administration of LPS induced higher amounts of microglial activation in the FA mouse model vs controls. We also observed an increase in oxidative damage in the form of 8-oxoguanine (8-oxo-G) and the DNA repair proteins MUTYH and PARP-1 in cerebellar microglia of FA mutant mice. We hypothesized that frataxin deficiency increases DNA damage and DNA repair genes specifically in microglia, activating them. siRNA-mediated frataxin knockdown in microglial BV2 cells clearly elevated DNA damage and the expression of DNA repair genes MUTYH and PARP-1. Frataxin knockdown also induced a higher level of PARP-1 in MEF cells, and this was suppressed in MUTYH-/- knockout cells. Administration of the PARP-1 inhibitor PJ34 attenuated the microglial activation induced by intracerebroventricular injection of LPS. The combined administration of LPS and angiotensin II provoke an even stronger activation of microglia and neurobehavioral impairment. PJ34 treatment attenuated the neurobehavioral impairments in FA mice. These results suggest that the DNA repair proteins MUTYH and PARP-1 may form a pathway regulating microglial activation initiated by DNA damage, and inhibition of microglial PARP-1 induction could be an important therapeutic target in Friedreich's ataxia.

## Introduction

Friedreich’s ataxia (FA) is the most commonly inherited recessive ataxia, with an incidence of approximately one in 50,000 [1, 2]. FA pathology includes neuro-degeneration, cardiomyopathy, and diabetes mellitus [3, 4, 5]. FA is caused by an inheritance of a trinucleotide repeat expansion of [GAA], which causes silencing and decreased expression of the frataxin gene that is primarily expressed in mitochondria [6, 7]. Frataxin is a small mitochondrial protein whose most biochemically defined role is as a sulfur donor for mitochondrial iron-sulfur cluster biogenesis. Frataxin deficiency may lead to oxidative stress, mitochondria dysfunction and inflammation [8–11]. Currently, there is no FDA-approved treatment for FA. Therefore, there is a need to develop an effective therapy for this disease. Recently, increased inflammatory metabolites and microglial activation have been observed in cerebella of FA mouse models and in patient cells. However, what incites the microglial to activate and produce the inflammatory metabolites is not understood.

A common cause of microglial activation in multiple sclerosis and other neuroinflammatory diseases is increased oxidative stress. Increased oxidative stress has been reported by multiple groups in multiple cellular and animal models of FA and in human FA patients [12–21]. ROS react with DNA in several ways, including by addition of hydroxyl radical or by abstraction of H atom, yielding multiple types of base lesions [16]. Both mitochondrial and nuclear DNA damage have been demonstrated in budding yeast with deletion of yeast frataxin homology YFH1 [17]. Nuclear DNA damage has been detected in peripheral blood cells from FA patients [18]. A remarkably higher level of 8-oxoguanine was found in urine samples from FA patients [19]. Mitochondrial DNA depletion caused by oxidative stress was also seen in FA heart samples [20]. Among all the DNA lesions, 8-oxoguanine is one of the most common oxidation products; this lesion can mismatch with adenine instead of cytosine and cause GC to TA transversions [21, 22, 23]. Multiple repair enzymes participate to repair 8-oxoG. Base excision repair (BER) is an important DNA repair pathway, which consists of a series of glycosylases that recognize and excise oxidized bases including 8-oxoG. MTH1, OGG1, and MUTYH constitute the 8-oxoG repair pathway [24, 25]. MTH1 hydrolyzes 8-oxo-dGTP and removes it from DNA pools, preventing incorporation of 8-oxoG into DNA. Furthermore, OGG1 excises 8-oxoG paired with cytosine. In the event of 8-oxoG•A mismatches, MUTYH can recognize and remove the adenine inserted opposite 8-oxoG, avoiding GC to TA transversions.

PARP-1, a well-known DNA-binding enzyme that catalyzes poly(ADP-ribosyl)ation on nuclear proteins, may also have an essential role in BER by recruiting repair enzymes to the damage sites and modifying chromatin structure. PARP-1 mainly repairs single-stranded DNA breaks, and MUTYH has been shown to recruit PARP-1 during BER by generating single-stranded DNA breaks [26, 27]. In our study, we found that 8-oxoG is increased during microglial activation induced by LPS treatment in FA transgenic mice. Higher levels of MUTYH and PARP-1 were also seen in activated microglia, and MUTYH knockout suppresses PARP-1 activation. This indicates that MUTYH is upstream of PARP-1 in this pathway. These results suggest that an important consequence of frataxin deficiency is DNA damage and consequent PARP-1 activation in microglia, which then could mediate neuroinflammatory neurodegeneration [28, 29]

Angiotensin II is a major vasoactive peptide in the renin-angiotensin system (RAS) [30, 31] and was recently shown to be pro-inflammatory [32, 33]. In our study, angiotensin II was combined with LPS, enhancing inflammation in FA mice. We found that combined administration of LPS and angiotensin II further exacerbated both the glial activation and behavioral deficits in FA mice vs. controls. In addition, PJ34 (a PARP-1 inhibitor) treatment attenuated both glial activation and the FA-specific behavior impairments. These results support the idea that a major downstream consequence of frataxin deficiency is increased DNA damage in microglia that stimulates microglial activation, and that PARP-1 inhibition is thus a rational FA therapy to reduce the neuroinflammatory burden.

## Materials and Methods

### Mice

Female and 6 month old endogenous frataxin-deficient KIKO mice [a kind gift from Dr. Pandolfo [34]] were housed in a vivarium maintained at 22–24°C and 40–60% relative humidity with a 12-h light/12-h dark cycle. Female and 6 month old C57BL/6J mice were used as the wild-type strain and were housed in the same vivarium. All experimental procedures were approved by the University of California Institutional Animal Care and Use Committee.

### Introcerebraventricular Injection of LPS

Mouse was placed into an induction chamber and anesthetized by 3% isoflurane. Then mouse was positioned on a stereotaxic frame. To maintain the anesthesia, a mask was used and isoflurane vaporizer was adjusted to 2%. LPS (0.5mg/kg) dissolved in PBS was injected into forth ventricle at coordinates: 3.0mm posterior to the bregma; 3.5 mm ventral to the surface of the skull; on the midline. Bupronorphin (0.1mg/kg) subcutaneous injection was given at the time of surgery and once every twelve hours until 72hours after the surgery. For control group, PBS injection was given at the same coordinates.

### Angiotensin II Subcutaneous Infusion

A miniosmotic pump 2007 (Alzet) containing angiotensin II (1mg/kg) was subcutaneously implanted into the back of the mouse. Control mice were infused with a same volume of PBS. Pumps were removed at eighth day after the implantation.

### PJ34 Treatment

One dose of PJ34 (5mg/kg) was given intraperitoneally at 30mins before stereotaxic injection of LPS. Two more dosages of PJ34 were given at 8 hour and 16 hour after the LPS injection. Control mice received same volume of PBS at same time points. To examine Iba-1 immunostaining level, mice were perfused at 24 hour after the surgery. For behavioral study, mice received one dose of PJ34 (5mg/kg) per day until 14 days after surgery.

### Immunohistochemistry

Mice were perfused with 4% paraformaldehyde in 0.1M PBS at one day or 7 day time point after stereotaxic surgeries. 10μm brain sections from cerebellum region were processed with cryostat. Fluorescence immunostainings were performed using Alexa Fluor 488- and Alexa Fluor 647- tagged secondary antibodies (Invitrogen). The following primary antibodies were used: Iba-1 antibody (1:100, Wako), CD11b (1:100, Abdserotec), MUTYH (1:500, Abcam), PARP-1 antibody (1:1000, Trevigen) and GFAP (1:500, Cell signaling). Primary antibodies were incubated overnight at 4°C followed by secondary antibody incubation two hours at room temperature. Fluorescent microscopy was performed with Retiga 2000R camera (Qimaging) mounted on a Nikon microscope (Nikon, USA). For statistical analyses, fluorescent immunostaining intensity was measured with ImageJ software (NIH) and cells were counted from 6 coronal sections per mouse.

### Immunostaining of 8-oxoG

Brain sections or cells on plate were fixed with 4% paraformaldehyde. Sections or cells were treated with 2NHCl for 5mins at room temperature after incubated with 100μg/ml RNase A for 1hr at 37°C. After neutralized with 1M Tris-base, sections or cells were incubated with 8-oxoG antibody (1:200, Trevigen) overnight followed by secondary antibody incubation.

### Cell Culture and Treatments

BV2 cell line was cultured with DMEM (Cellgro) supplemented with 10% fetal bovine serum (JR-Scientific). As described in reference [35], Mutyh -/- mouse embryonic fibroblast (MEF) cell lines were created by crossing Mutyh +/- littermates and spontaneously immortalized. Presence of the targeted Mutyh deletion insert in exon 6 was verified via PCR analysis. The 535 amino acid human MUTYH protein isoform (Ref Seq NP_001041636.1 encoded by the type 1 (alpha3) mRNA isoform NM_001048171.1) was cloned into a pcDNA3.1 vector (Invitrogen) and was transiently transfected with attractene transfection reagent (Qiagen) into Mutyh-/- MEFs. Cells were treated with 400–800 μg/mL G418 antibiotic, and after 7–14 days, single resistant colonies were selected with cloning cylinders. Mouse MEF cell line and MUTYH-/- MEF cell lines were cultured with DMEM/F12-Glutamax (Gibco) with 10% fetal bovine serum and 0.1% MEM NEAA(Gibco). ON-TARGETplus Mouse Frataxin siRNA #5 (Target Sequence: GGACCUACGUGAUCAACAA), #6 (TargetSequence: CGAGACAGCGUAUGAAAGA), #7 (Target Sequence: GGAGGGAACCGAUCGUAA), and ON-TARGETplus Mouse Frataxin siRNA Smart Pool (Dharmacon) or non-target siRNA pool (Thermo Scientific) were used to transfect cells with Lipofectamine RNAiMAX (Invitrogen). MEF cells were treated with 250μM MNNG (Chem Service) with different duration: 30mins, 2hrs and 4hrs.

### Western Blot Analysis

Mouse cerebellar tissues and cell pellets were homogenized with a cell lysis buffer (Cell Signaling) with Halt phosphatase inhibitor (Thermo-Fisher), complete protease inhibitors (Roche) and PSMF (Sigma-Aldrich). The supernatant was recovered by centrifugation (16,000g, 15mins). Protein concentration was assayed by the Bradford protein assay system (Bio-rad). Twenty micrograms of lysates were loaded into 4–12% Bis—Tris gels (Invitro-gen). Electrophoresis was carried out according to the manufacturer’s recommendations. Following electrophoresis, the proteins were transferred to nitrocellulose membranes by the iBlot device (Invitrogen), blocked with an Odyssey blocking buffer (LI-COR Biotechnology) for 1 h. Membranes were incubated overnight with the following primary antibodies in blocking buffer: Iba1(Wako), CD11b(Abcam), MUTYH(Abcam), PARP-1(Trevigen), Frataxin (Santa Cruz), Actin(Abcam), AT1(Abcam), Tubulin (Abcam). Subsequently, the membranes were incubated with a corresponding pair of IRDye 680CW and IRDye 800CW-coupled secondary antibodies (LI-COR). Proteins were visualized with the Odyssey infrared imager and software (LI-COR) according the manufacturer’s instruction.

### TUNEL Assay

TUNEL staining was performed using Promega DeadEnd Kit. Briefly, sections were fixed with 4% paraformaldehyde. After equilibration, sections were incubated with TdT reaction mix for 1hr at 37°C. Stop the reaction with 2xSSC and then mount and analyze. For double staining with NeuN, before TUNEL staining, sections were incubated with NeuN antibody (Milipore) followed by Alexa Fluor 647- tagged secondary antibody.

### Level Beam Test

The level beam apparatus consisted of 1 meter beams with 21 mm, 12 mm or 9 mm width, resting 50 cm above the table top on two poles. A black box was placed at the end of the beam as the finish point. Nesting material from home cages was placed in the black box to attract the mouse to the finish point. A lamp (with 60 watt light bulb) was used to shine light above the start point and served as an aversive stimulus. The time to cross the center 80 cm was recorded. Before testing, each mouse was trained three times on each size of beams. The time of three crossings were recorded for each mouse and analyzed to get quantitative data. A code was given to every mouse to cover its identity, so researcher was blind to the experiment.

### Treadscan Analysis

Treadmill device was purchased from CleverSys. Briefly, the treadmill consisted of a motor-driven transparent treadmill belt with an angled mirror mounted below. A high-speed digital video camera was mounted to record the ventral view of the treadmill belt reflected off the mirror; digital video images of the underside of the mouse were recorded at 100 frames/s. TreadScan software (CleverSys) identified each individual paw of the mouse in each frame as it walked on the treadmill. Before testing, each mouse was trained on the treadmill with gradually increasing speed from 9cm/s to 18cm/s. For testing, mice were walking at 15cm/s. The video images were recorded and analyzed with TreadScan software (CleverSys). Gait parameters were calculated for quantitative data analyses. Each mouse received a code to cover its identity, so researchers were blind to the experiment.

## Results

### Intracerebroventricular Injection of LPS Induced Obvious Neuroinflammation in FA Mouse Model Cerebellum vs Controls

**Fig 1:**
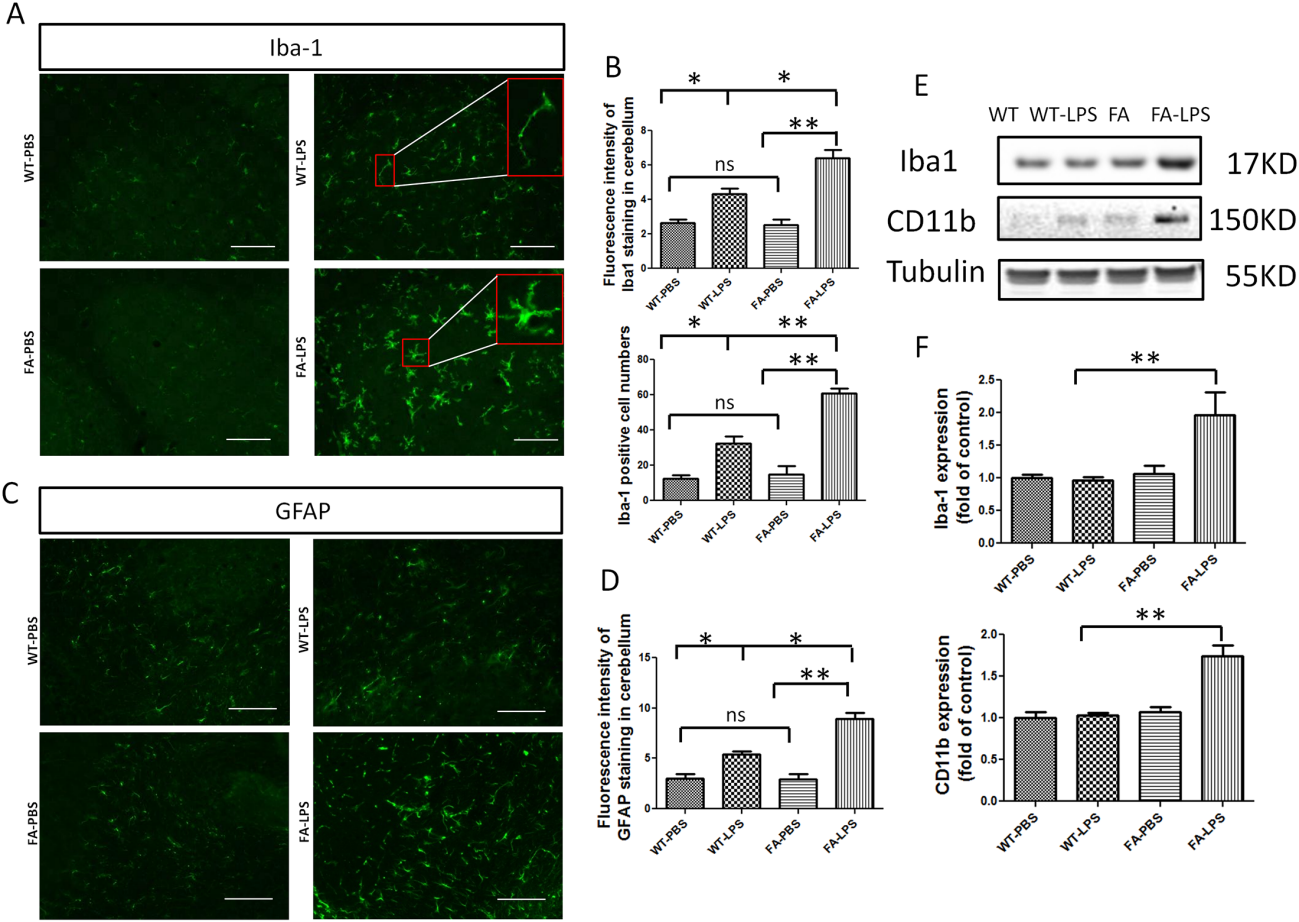
Introcerebraventricular injection of LPS induced more microglia activation and astrocyte activation in FA mice. (A) FA mice receiving introcerebraventricular injection of LPS exhibited more Iba-1 immunoreactivity compared with WT mice receiving introcerebraventricular injection of LPS, and WT or FA mice received introcerebraventricular injection of vehicle (PBS). Scale bar: 20μm. (B) Quantitative measurement of Iba-1 immunoreactivities showed FA mice treated with LPS had significantly higher Iba-1 staining intensity compared with other FA mice only receiving PBS, and WT mice with or without LPS treatment. FA mice treated with LPS had significantly more Iba-1 positive staining microglia compared with other groups of mice. (C) FA mice receiving introcerebraventricular injection of LPS exhibited more GFAP immunoreactivity compared with WT mice received introcerebraventricular injection of LPS and WT, or FA mice receiving introcerebraventricular injection of vehicle (PBS). Scale bar: 20μm. (D) Quantitative measurement of GFAP immunoreactivity showed FA mice treated with LPS had significantly higher Iba-1 staining intensity compared with other FA mice only receiving PBS and WT mice with or without LPS treatment. Data are expressed as mean ± s.e.m. (t test, *p<0.05, ** *p*<0.01, n = 4). (E) Western blot analysis of Iba-1 and CD11b showed expression level of Iba-1 and CD11b are increased in FA cerebellum after LPS introcerebraventricular injection. (F) Quantitative measurement of Western blot bands showed cerebellums of FA mice treated with LPS had significantly higher expression level of Iba-1 and CD11b. Data are expressed as mean ± s.e.m. (t test, ** *p*< 0.01, n = 5).

Given the observation of inflammatory effects caused by frataxin deficiency [10,11], we tested the consequences of a direct inflammatory insult to brains of FA mice. LPS was stereotactically injected into the forth ventricle of FA mice and wild type mice. At one day or one week time points, respectively, mice were sacrificed and perfused, and brain sections from cerebellum were processed and stained with Iba-1 antibody. Fluorescent images showed that there are obviously more microglia in the cerebella of FA mice compared to WT treated with LPS injection and also more in LPS-treated FA mice compared to PBS-treated FA mice (Fig 1A). Most microglia in the cerebellum of FA mice showed distinctive activated amoeboid morphology: i.e. swollen cell bodies with shortened processes. Quantitative analyses of fluorescence intensity performed with NIH ImageJ software showed that there was significantly more Iba-1 immunoreactivity in the cerebellum of FA mice. Consistent with this, cell counts also revealed significantly more Iba-1 positive cells in the cerebellum of FA mice than controls (Fig 1B). Thus there was significantly more microgliosis in FA mice vs. controls after stimulus. GFAP was also used as a marker of astrocytes at one week after LPS injection. There was an obvious increase in astrocytes in FA mice compared to WT mice treated with LPS injection or FA mice treated with PBS (Fig 1C). Quantitative analyses of fluorescence intensity with ImageJ showed significantly more GFAP immunoreactivity in the cerebellum of FA mice (Fig 1D). Thus there was significantly more astrogliosis in LPS-treated FA mice. Iba-1 and GFAP immunoreactivities were significantly increased in WT mice treated with LPS compared with WT mice treated with PBS. There was not a significant difference between WT mice treated with PBS and FA mice treated with PBS (Fig 1B and 1D). Western blots of microglial-specific markers Iba-1 and CD11b confirmed that there was more microglial activation in FA mice treated with LPS. Western blots showed that the expression level of Iba-1 and CD11b was significantly increased in FA mice treated with LPS introcerebroventricular injection (Fig 1E and 1F). These results show that FA mice have increased microglial activation in the brain.

### DNA Damage and MUTYH and PARP-1 Are Increased Specifically in Microglia of FA Mice

**Fig 2:**
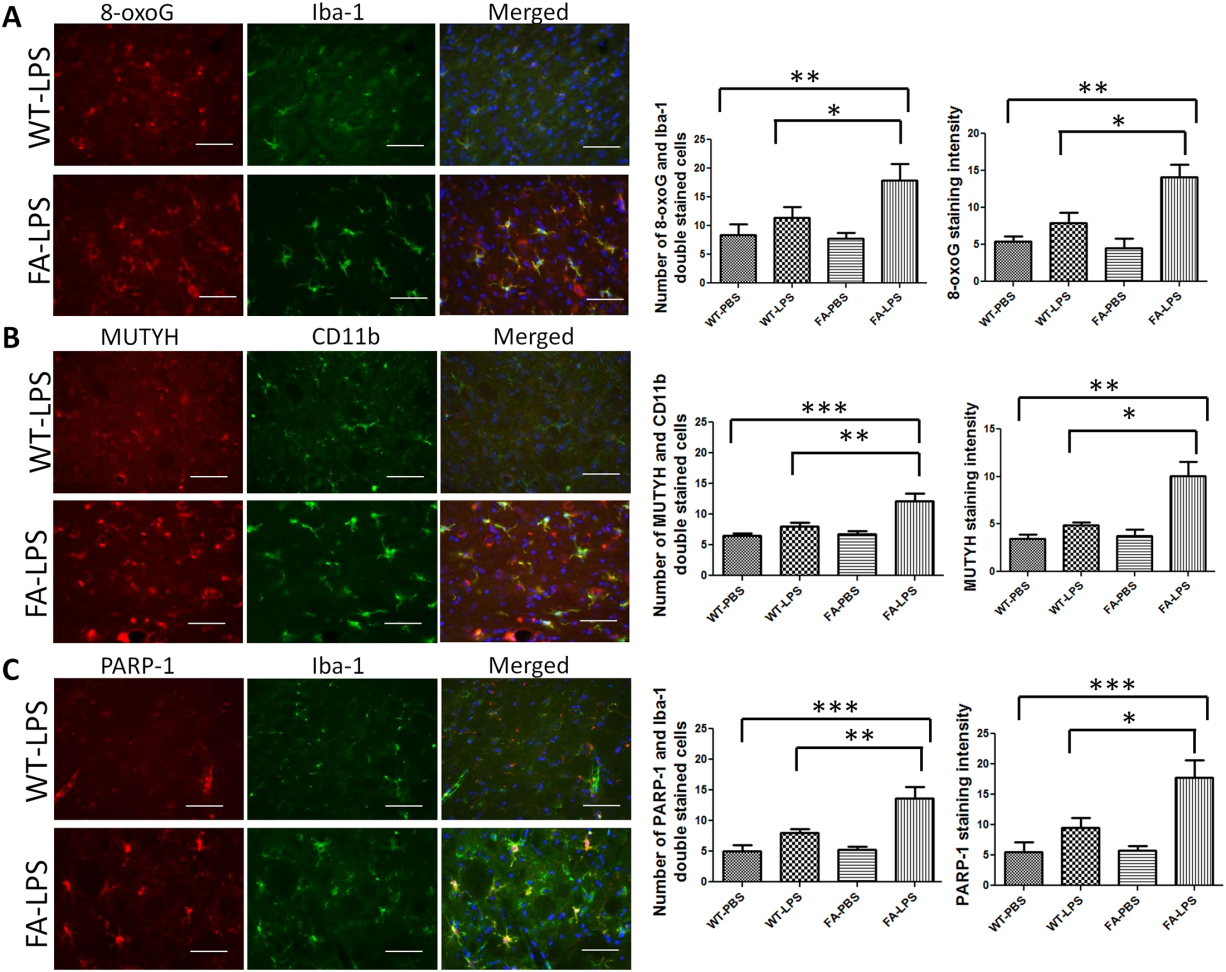
8-oxoG, PARP-1 and MUTYH are increased in cerebellum of FA mice treated with introcerebraventricular injection of LPS compared with wild-type mice received the same treatment. (A) DNA damage marker, 8-oxoG was double stained with Iba-1. Cerebellum of FA mice has more 8-oxoG immunoreactivities and 8-oxoG/Iba-1 double-stained cells. (B) MUTYH was double stained with CD11b. Cerebellum of FA mice has more MUTYH immunoreactivity and MUTYH/CD11b double-stained cells. (C) PARP-1 was double stained with Iba-1. Cerebellum of FA mice has more PARP-1 immunoreactivity and PARP-1/Iba-1 double stained cells. Scale bar:10μm. Data are expressed as mean ± s.e.m. (t test and one way ANOVA, * *p*< 0.05, ** *p*<0.01, *** *p*<0.001, n = 4).

To investigate the possible mechanism of microglial activation in FA mice, we examined a possible inciting insult, i.e. oxidative DNA damage, using the 8-oxoG antibody, the resultant response using MUTYH and PARP1 antibodies. and the overlap with microglia using the the Iba-1 and CD11b antibodies (Fig 2). It was clear that LPS-treated FA mice had higher levels of 8-oxoG, MUTYH, and PARP-1 compared to LPS-treated WT mice, and that this signal was specific to microglia co-stained with Iba-1 and CD11b (Fig 2A–2C). These results indicate that intracerebroventricular LPS treatment specifically induces oxidative DNA damage in microglia and elevates the level of the DNA repair genes MUTYH and PARP-1 under the conditions of *in vivo* frataxin deficiency.

### Frataxin Knockdown Increased Oxidative DNA Damage and the Expression Level of MUTYH and PARP-1 in a Microglial Cell Line

**Fig 3:**
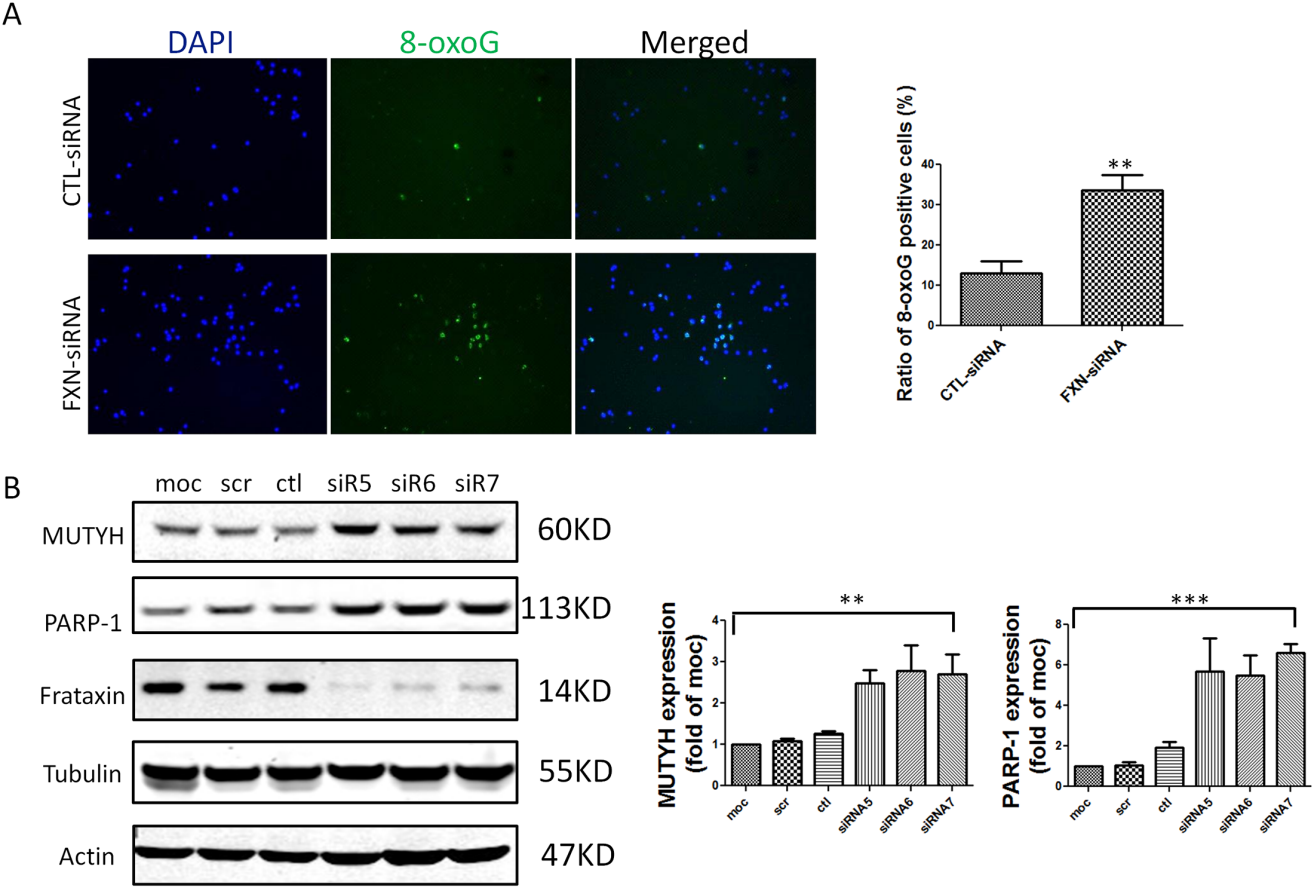
8-oxoG, MUTYH and PARP-1 are increased in frataxin knockdown BV2 cells. (A) 8-oxoG was labeled in BV2 cell culture. BV2 cells treated with frataxin siRNA have more 8-oxoG immunoreactivity compared to BV2 cells treated with control siRNA. Data are expressed as mean± s.e.m. (t test, ** *p* < 0.01, n = 3) (B) Western-blot of MUTYH and PARP-1 in BV2 cells showed that BV2 cells treated with frataxin siRNAs (siR5, siR6 and siR7) expressed more MUTYH and PARP-1 compared to BV2 cells treated with control siRNA. Data are expressed as mean± s.e.m. (one way ANOVA, ** *p* < 0.01, *** *p*<0.001, n = 3).

To test the cell autonomy of the above results, i.e. a frataxin-deficient effect on oxidant stress and MUTYH and PARP1, we knocked down frataxin in the BV2 microglial cell line (Fig 3). Similar to the situation in living mice, frataxin knockdown in microglial BV2 cells caused higher 8-oxoG levels and higher MUTYH and PARP-1 (Fig 3). Thus frataxin deficiency causes DNA damage and induces DNA repair genes in the microglial context. In the animal model, the stain for 8-oxoG, MUTYH, and PARP-1 specifically overlapped with microglia and not other cells (Fig 2A–2C).

### Relationship between and Regulation of PARP-1 by MUTYH

**Fig 4:**
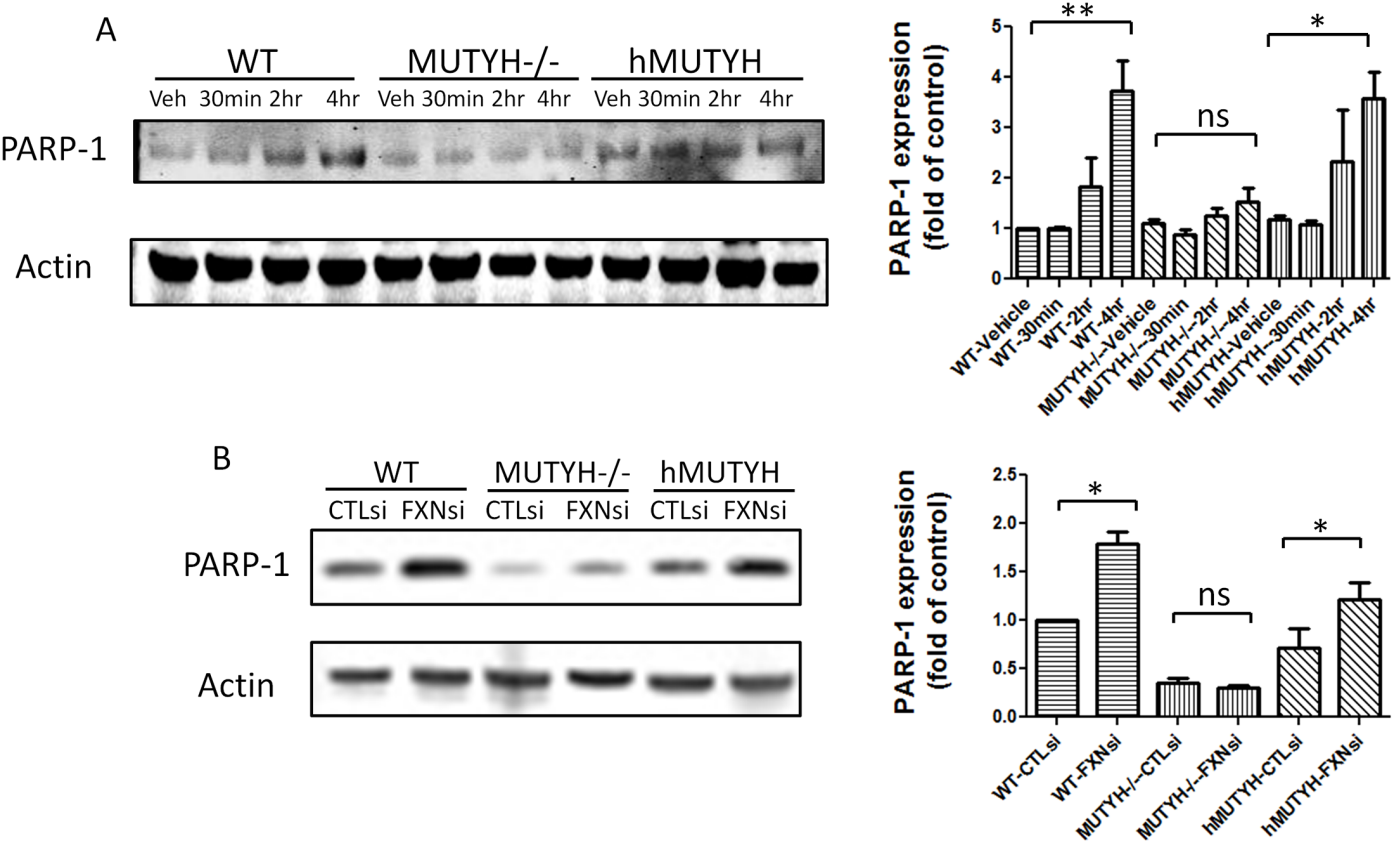
PARP-1 is less inducible in MUTYH-/-MEF cell by MNNG and frataxin siRNA. (A) Western blot of PARP-1 in MEF cells showed that MNNG treatment induced higher level of PARP-1 in WT MEF cells and in MUTYH-/- MEF cells expressing human MUTYH (labeled as hMUTYH) compared to MUTYH -/- MEF cells. Data represent the densitometry results of the western blots. Data are expressed as mean± s.e.m. (one way ANOVA, * *p*<0.05, ** *p* < 0.01, n = 3)(B) Western blot of PARP-1 in MEF cells showed that frataxin siRNA transfection induced higher level of PARP-1 in WT MEF cells and in MUTYH-/- MEF cells expressing human MUTYH (labeled as hMUTYH) compared to MUTYH -/- MEF cells. Data represent the densitometry results of the western blots. Data are expressed as mean± s.e.m. (t test, * *p*<0.05, n = 3).

Previous work from other groups suggested that MUTYH initiates BER-activated PARP-1 dependent cell death pathways by generating single strand breaks [26, 27]. MUTYH encodes adenine DNA glycosylase which removes the adenine inserted opposite 8-oxoG, leaving single-strand breaks [36]. PARP-1 binds to single-strand breaks and becomes activated [37]. We used a PARP-1 inducer MNNG to treat wild-type MEF cells, MUTYH-/- MEF cells, and MUTYH-/- MEF cells expressing human MUTYH. PARP-1 was MNNG-inducible in wild-type MEF cells and MUTYH -/- MEF cells co-expressing human MUTYH, but not in MUTYH-/- MEF cells (Fig 4A). Thus induction of PARP-1 depends on functional MUTYH (Fig 4A). To further confirm PARP-1 and MUTYH are associated in frataxin deficiency, Wild-type MEF cells, MUTYH-/- MEF cells, and human MUTYH-expressing MEF cells were treated with frataxin siRNA. Frataxin knockdown induced more PARP-1 expression compared to control siRNA in WT MEF cells and human MUTYH-expressing MEF cells (Fig 4B). Frataxin knockdown caused no significant difference in PARP-1 level in MUTYH-/- MEF cells (Fig 4B). These results suggest that MUTYH and PARP-1 form a pathway repairing DNA damage caused by frataxin deficiency in microglia.

### PARP-1 Inhibitor PJ34 Attenuates Microglial Activation in Cerebellum of FA Mice Treated with LPS

**Fig 5:**
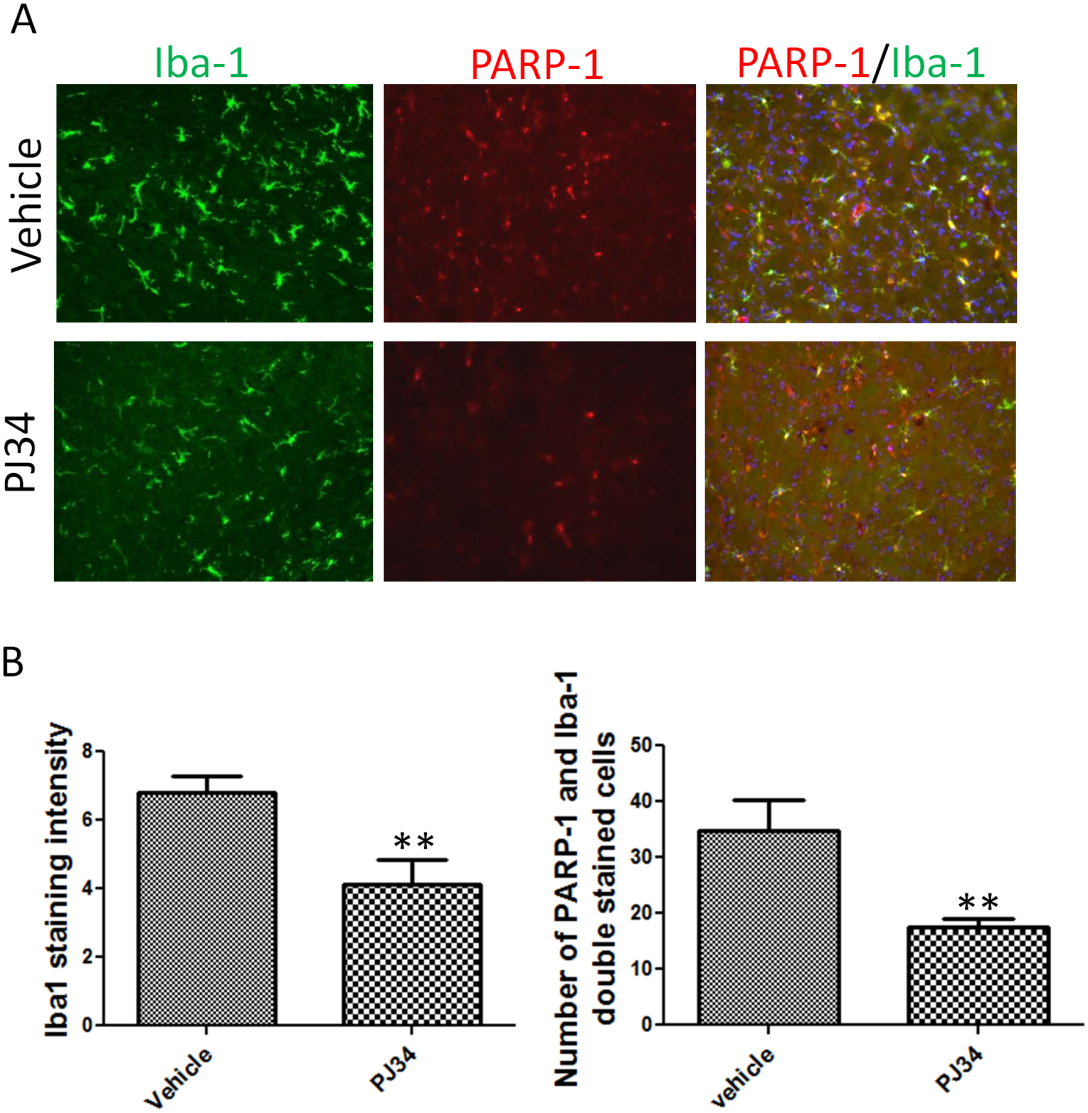
PARP-1 inhibitor, PJ34 attenuated microglia activation in cerebellum of FA mice treated with LPS. (A) Both Iba-1 and PARP-1 immunoreactivities are attenuated in cerebellum of LPS treated FA mice by administration of PARP-1 inhibitor, PJ34. Merged PARP-1 and Ib1-1 staining showed less double stained cells with PJ34 treatment compared to vehicle. (B) Quantification of Iba-1 staining intensity and cell count of PARP-1/Ib1-1 doubled cells showed significant differences between PJ34 treated group and Vehicle treated group. Data are expressed as mean± s.e.m. (t test, ** *p*<0.01, n = 5).

PARP-1 increases inflammatory gene activity and microglial activation [28, 29]. In both a microglial cell line and FA mice, we found that frataxin deficiency leads to oxidative DNA damage and higher levels of PARP-1. To test the possibility that the activation of PARP-1 induced by DNA damage is responsible for microglial activation caused by frataxin deficiency, we used PJ34, a PARP-1 inhibitor, to treat LPS-exposed FA mice. Controls were LPS-exposed FA mice receiving PBS. Brain tissues were collected after one day of the LPS stereotactic injection and three doses of PJ34 or PBS. Sections from the cerebellum of FA mice were stained with Iba-1. Iba-1 intensity was reduced in the PJ34-treated group compared to the vehicle group (Fig 5A). Counting of PARP-1/Iba-1 double stained cells showed significantly less PARP-1/Iba-1 double stained cells in the PJ34 treatment group (Fig 5B). This result suggests that the activity of PARP-1 is essential to activate microglia. Taken together, our results suggest that the frataxin deficiency induces oxidative DNA damage that activates PARP-1 and thus microglia, and that PARP-1 inhibition attenuates this process of frataxin-dependent microglial activation.

### Angiotensin II Treatment Exacerbates Glial Activation and Causes Neuronal Cellular Damage

**Fig 6:**
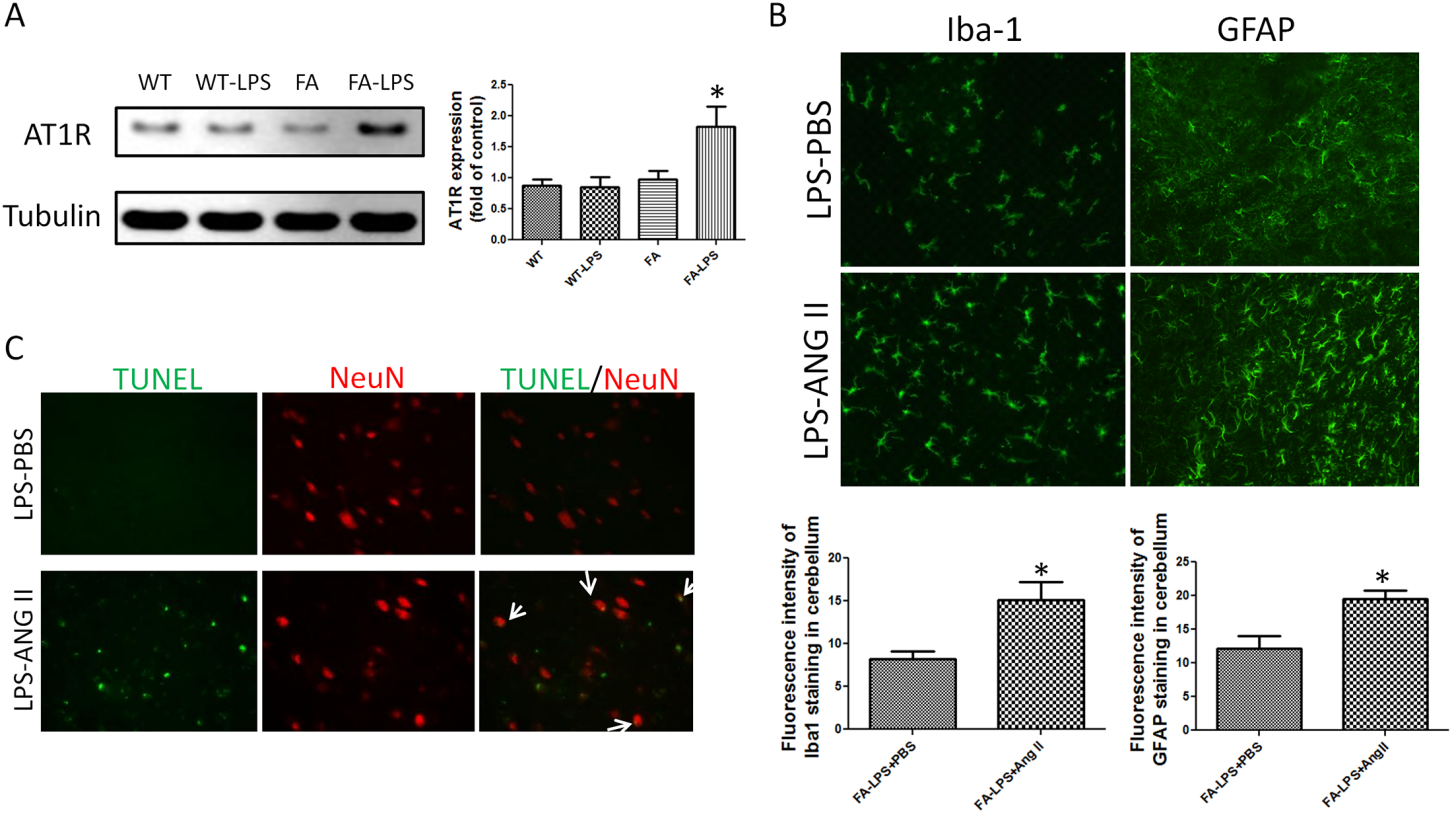
Angiotensin II treatment exacerbates microglial activation and astrocyte activation and causes neuronal cellular damage. (A) Western blot of angiotensin II type 1 receptor (AT1R) showed that LPS treatment induced more AT1R expression in cerebellum of FA mice compared to wild type mice. Data are expressed as mean± s.e.m. (one way ANOVA, * *p*<0.05, n = 5). (B) Angiotensin II infusion increased microglia activation and astrocyte activation in cerebellum of LPS treated mice showed by Iba-1 and GFAP staining. Quantitative analyses of Iba-1 and GFAP staining intensity showed significant differences between angiotensin II infusion group and PBS infusion group. Data are expressed as mean± s.e.m. (t test, * *p*<0.05, n = 5). (C) Angiotensin II infusion exacerbated neuronal damage showed with TUNEL staining. More TUNEL immunoreactivity was seen in cerebellum of FA mice treated with LPS combined with angiotensin II infusion compared to FA mice treated with LPS combined with PBS infusion. Partial of TUNEL staining were overlapped with NeuN staining. Angiotensin II infusion group had more TUNEL/NeuN doubled stained cells.

Western blots of angiotensin II type 1 receptor (AT1R) showed a higher expression level of AT1R in FA mice treated with LPS compared to control mice, suggesting some frataxin-dependence on the angiotensin response (Fig 6A). Angiotensin II was administered to evaluate the function of AT1R. Immunostaining of Iba-1 and GFAP showed that FA mice receiving both combined LPS and angiotensin II treatment had significantly more immunoreativity of Iba-1 and GFAP compared to FA mice treated with LPS injection (Fig 6B). TUNEL staining showed that there were significantly more TUNEL positive cells in the cerebellum of FA mice treated with combined LPS injection and angiotensin infusion compared to FA mice treated with LPS injection and PBS infusion (Fig 6C). The overlap of NeuN staining and TUNEL staining indicates that part of TUNEL positive cells are neuronal (Fig 6C). This result suggested that combined LPS injection and angiotension II infusion caused neuronal cell death which was not seen in FA mice treated with LPS and PBS infusion alone.

### PJ34 Attenuates Behavioral Impairments Caused by Combined LPS Injection and Angiotensin II Infusion in FA Mice

**Fig 7:**
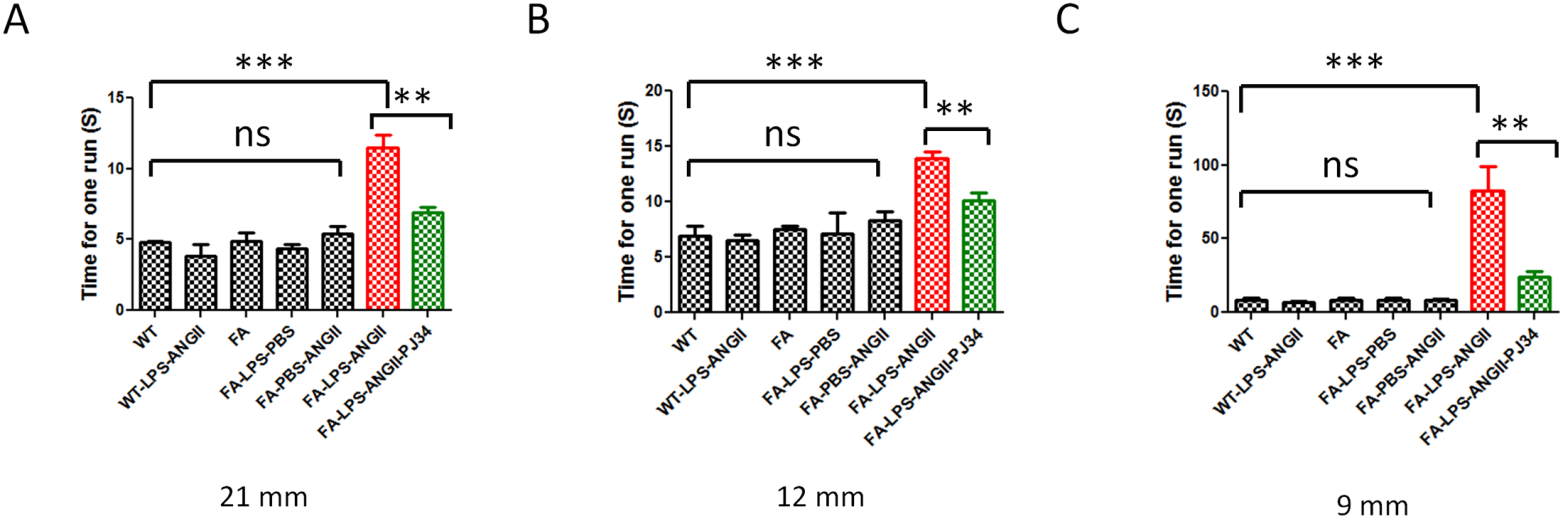
Level beam tests showed that PJ34 treatment attenuates behavior impairments caused by combined LPS and angiotensin II treatment in FA mice. Level beam tests showed that FA mice treated with LPS combined angiotensin II infusion walked significantly slower than other groups on (A) 21mm, (B) 12mm and (C) 9mm level beams. PJ34 treatment attenuated the behavior impairments caused by LPS and angiotensin II combined treatment. After receiving PJ34 treatment, FA mice treated with LPS and angiotensin II infusion walked significantly faster than FA mice treated with LPS and angiotensin II infusion but not receiving PJ34 on the level beams. Data are expressed as mean± s.e.m. (t test or one way ANOVA, * *p*<0.05, ** *p*<0.01, *** *p*< 0.001, n = 5).

**Fig 8:**
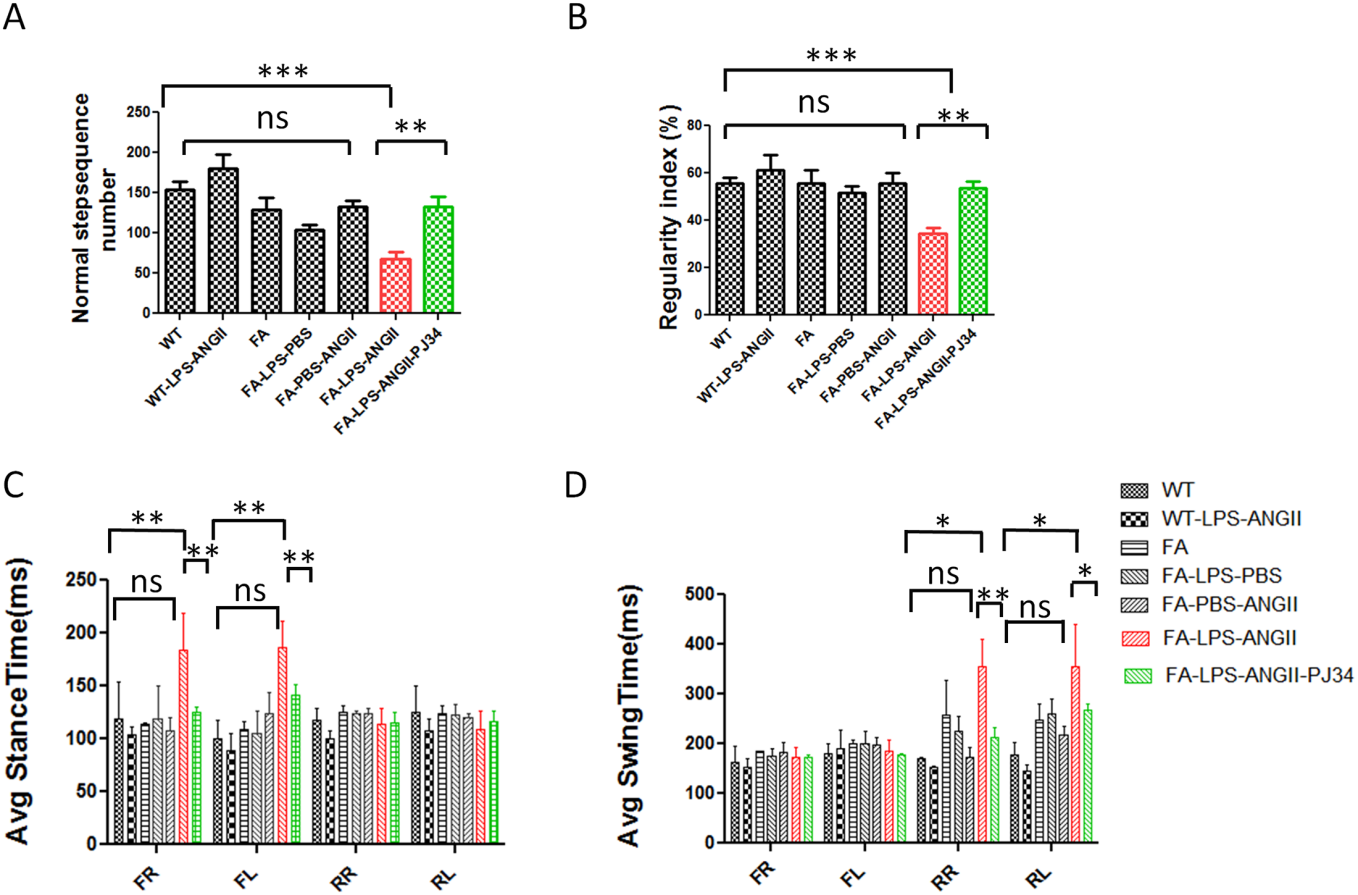
Treadscan tests showed that PJ34 treatment attenuates behavior impairments caused by combined LPS and angiotensin II treatment in FA mice. Treadscan tests showed that FA mice treated with LPS combined angiotensin II infusion had significantly less normal stepsequence numbers than other groups (A). FA mice treated with LPS combined angiotensin II infusion had significantly smaller regularity index compared to other groups (B). LPS combined angiotensin II infusion on FA mice increased average stance time of front feet (C) and average swing time of rear feet (D) compared to untreated mice and LPS or ANG II only treated FA mice. PJ34 treatment reversed the effects of LPS combined with angiotensin II infusion. LPS injection only or angiotensin II infusion only didn’t cause any behavior deficit on either wild type mice or FA mice. Data are expressed as mean± s.e.m. (t test or one way ANOVA, * *p*<0.05, ** *p*<0.01, *** *p*< 0.001, n = 5).

As combined LPS injection and angiotensin II infusion caused neuronal cell death, we tested whether the death was sufficient to cause neurobehavioral deficits. Neurobehavioral outcomes and rescue were measured by level beam and Treadscan. FA mice treated with combined LPS and angiotensin II walked significantly slower on different sizes of level beams (Fig 7). The narrowest level beam (9mm) showed the most significant difference (Fig 7C). LPS injection or angiotensin II infusion alone caused trends but no significant behavioral impairment. There was no difference between WT and FA untreated groups, suggesting that frataxin deficiency alone was not sufficient to cause a defect, but that LPS plus angiotensin plus frataxin deficiency were required (Fig 7). Step sequence number and gait regularity are an integrative and consistent measure of animal models of ataxia [38,39]. FA mice treated with combined LPS and angiotensin II had the lowest Treadscan step sequence number and regularity index compared to other groups (Fig 7B). This gait problem may be caused by dysregulation of stance time and swing time in those mice. FA mice treated with LPS plus angiotensin II had significant longer stance time on front feet and significant longer swing time on rear feet (Fig 8). There is no difference between WT untreated group to WT treated with LPS plus angiotensin II, but a frataxin-deficient genetic background is required to cause the behavioral outcome (Fig 8). PJ34 treatment significantly rescued the behavioral impairments on FA mice caused by combined LPS injection and angiotensin II infusion (Fig 8). Thus PARP1 attenuation provides a functional benefit in the frataxin-deficient state. PARP-1, an important initiator of microglial activation, could be a potent therapeutic target of FA.

## Discussion

### Frataxin Deficiency Causes Neuroinflammation

In our previous study, frataxin-knockdown caused inflammatory death of Schwann cells with strong stimulation of cytokines and prostaglandins, and a rescue by anti-inflammatory drugs [10]. In two mouse models of FA and in FA patient cells, we showed the elevation of COX2-mediated prostaglandin synthesis and excess microglial activation [11]. These results suggest that frataxin deficiency causes neuroninflammation, but don't suggest a mechanism how and why. Microglia are the primary resident immune cells in CNS, whose activation is a key step in neuroinflammation [40]. Microglia in FA mice appear to be in a resting stage, similar to microglia in wild-type mice. However, intracerebroventricular treatment of LPS revealed that microglia in FA mice are in a more activated state than microglia in wild-type mice. Since there was excessive microglial activation in the complex milieu of the frataxin-deficient cerebellum, this brings up the question of whether this occurs cell autonomously or not.

### Frataxin-Deficiency Leads to Microglial Damage *In Vivo* and *In Vitro*

Microglial activation is an important cause of neuroinflammation but not all causes of microglial activation are known [40, 41, 42, 43]. We observed microglial activation *in vivo* in frataxin-deficient FA mice, and we showed that this was also a property of frataxin-deficient BV2 microglial cells *in vitro*, thus frataxin deficiency cell-autonomously activates microglia. One possible mechanism follows: frataxin deficiency increases oxidative stress to DNA, and thus oxidative DNA damage, which in turn induces the MUTYH enzyme to repair the oxidative damage, and then PARP-1 is induced as a downstream consequence [44]. This idea was confirmed by our results showing that induction of MUTYH and PARP-1 was frataxin-dependent (Fig 3), and that the frataxin-dependent induction of PARP-1 was MUTYH-dependent (Fig 4). Another less likely possibility is the following: frataxin's identified physiological role is as an iron-sulfur cluster biogenesis protein, and MUTYH is a 4Fe-4S cluster containing protein. Thus, frataxin deficiency could cause a 4Fe-4S cluster deficit and cause a consequent induction of MUTYH and downstream PARP-1; however, the result is still increased PARP-1, which increases microglial activation. Thus, PARP1 inhibitory strategies like PJ34 are still relevant under this second hypothetical model.

### Mechanistic Considerations of PARP-1 Inhibition

If PARP-1 activation is downstream of frataxin deficiency and upstream of microglial activation that incites the neuroinflammatory process, then inhibiting of PARP-1 with PJ34 is a reasonable strategy to reduce frataxin-dependent neuroinflammation. PJ34 is a relatively specific inhibitor of PARP-1. It is approximately 10,000 times more potent than the prototypical PARP inhibitor, 3-AB [45]. PJ34 has demonstrated widespread therapeutic activity in suppressing the inflammatory response in models of ischemia and autoimmune disease [45, 46, 47]. In our study, the anti-inflammatory and neuroprotective effect of PJ34 support the potential of PARP-1 being an important therapeutic target of FA. Some studies have shown effects of PJ34 on other targets than PARP1, including serine/threonine kinases, Pim1/2 and p21 [48, 49]. The low dose of PJ34 (5mg/kg) used in the present study is much lower than the commonly used doses of 10mg/kg to 20 mg/kg [45, 46, 47], which decreases the likelihood that PJ34-mediated protection is the result of off-target effects.

### Angiotensin II Type 1 Receptor May Have a Contributory Role in Microglial Activation in FA

The renin-angiotensin-aldosterone (RAS) regulates blood pressure and body fluid homeostasis [30, 31]. Angiotensin II, which is generated by renin and angiotensin-converting enzyme from cleaving angiotensinogen, is the major effector molecule in RAS. Angiotensin II has two types of receptors: type 1(AT1R) and type 2 (AT2R). Recent evidence shows that angiotensin II is involved in neuroinflammation and microglial activation in the CNS. AT1R is mainly responsible for the inflammatory effects of angiotensin II [32, 33]. AT1R is highly expressed on microglia, and its expression increases during microglial activation [33, 50]. We observed that LPS induced more AT1R in FA mice than controls (Fig 6). Angiotensin II treatment increased microglial activation and astrocyte activation, suggesting that the role of AT1R in FA is related with neuroinflammation. Antagonists of AT1R have been shown to be neuroprotective in the animal models of Alzheimer disease [51], Parkinson’s disease [52] and multiple sclerosis [33]. Because of the frataxin-dependent overexpression of ATR1R in FA mice undergoing neuroinflammatory challenge, this suggests that ATR1R antagonists may be a supplementary rational therapeutic strategy in FA in addition to PJ34-mediated PARP-1 suppression.

## Conclusion

We found that introcerebral LPS treatment elicits more microglial activation in FA mutant mice brains than controls. We also identify a novel mechanism for microglial incitement as a consequence of frataxin-deficiency, i.e. that frataxin deficiency causes increased 8-oxoG DNA damage specifically in microglia, inducing MUTYH and consequently PARP-1, which in turn induces microglial activation that promotes neuro-inflammation. There are other possible mechanisms but this is the simplest consistent with our data. The combined treatment of LPS and angiotensin II provided a proinflammatory stimulus that induced neurobehavioral deficits and neurodegeneration specifically in FA mice and not controls. This treatment also provided functional criteria for evaluation of drugs. The PARP-1 inhibitor PJ34 attenuated glial activation and behavioral deficits in FA mice. These results suggest that microglial PARP-1 activation is an important consequence of frataxin deficiency and is a relevant therapeutic target for FA. We observed a frataxin-dependent excess in AT1R expression in FA mice as well, which supports the idea that anti-AT1R antagonists could also rationally benefit FA patients. Thus this study supports a novel neuroinflammatory mechanism downstream of frataxin depletion that implicates microglial-specific DNA damage, MUTYH and PARP-1 induction, and suggests that therapeutic amelioration of this pathway may be of therapeutic benefit.

## Supporting Information

S1 File Fig A. The flourescence intensity of iba1 staining in cerebellum is shown. Fig B. The fluorescence intensity of GFAP staining in cerebellum is shown. Fig C. The number of double-stained PARP-1 and iba-1 double-stained cells in cerebella are shown. Fig D. The number of double-stained 8-oxoG and iba-1-double-stained cells in cerebella are shown. Fig E. The relative PARP-1 expression as Fold-change of Mock-transfected cells with 3 frataxin knockdown constructs is shown. Fig F. The relative MUTYH expression as Fold-change of Mock-transfected cells with 3 frataxin knockdown constructs is shown. Fig G. The PARP-1 expression in MUTYH-/- cells and frataxin KD cells and the combination are shown. Fig H. The effects of MNNG damage on PARP1 expression in the context of presence or absence of MutYH are shown. Fig I. The intensity of iba1 staining in cerebella of FA model mice dosed with LPS, and LPS+ angiotensin are shown. Fig J. The time to run the beam in seconds is shown for LPS and angiotensin treated mice with or without PJ34. Fig K. The treadscan regularity of gait index, in the context of LPS and Angiotensin treatment is shown. Fig L. The average stance time in the different treatment groups are shown. B&W bars refer to the same treatment groups as in S11, red bars are LPS+Ang treatment, green bars are LPS+Ang+PJ34.
(ZIP)
